# Effect of the vitamin B12-binding protein haptocorrin present in human milk on a panel of commensal and pathogenic bacteria

**DOI:** 10.1186/1756-0500-4-208

**Published:** 2011-06-21

**Authors:** Henrik R Jensen, Martin F Laursen, Dorte L Lildballe, Jens B Andersen, Ebba Nexø, Tine R Licht

**Affiliations:** 1 National Food Institute, Mørkhøj Bygade 19, Technical University of Denmark, DK-2860 Søborg, Denmark; 2 Aarhus University Hospital, Norrebrogade 44, Department of Clinical Biochemistry, DK-8000 Aarhus, Denmark

## Abstract

**Background:**

Haptocorrin is a vitamin B12-binding protein present in high amounts in different body fluids including human milk. Haptocorrin has previously been shown to inhibit the growth of specific *E. coli *strains, and the aim of the present study was to elucidate whether the antibacterial properties of this protein may exert a general defense against pathogens and/or affect the composition of the developing microbiota in the gastrointestinal tracts of breastfed infants.

**Findings:**

The present work was the first systematic study of the effect of haptocorrin on bacterial growth, and included 34 commensal and pathogenic bacteria to which infants are likely to be exposed. Well-diffusion assays addressing antibacterial effects were performed with human milk, haptocorrin-free human milk, porcine holo-haptocorrin (saturated with B-12) and human apo-haptocorrin (unsaturated). Human milk inhibited the growth of *S. thermophilus *and the pathogenic strains *L. monocytogenes LO28, L. monocytogenes 4446 *and *L. monocytogenes 7291*, but the inhibition could not be ascribed to haptocorrin. Human apo-haptocorrin inhibited the growth of only a single bacterial strain (*Bifidobacterium breve)*, while porcine holo-haptocorrin did not show any inhibitory effect.

**Conclusions:**

Our results suggest that haptocorrin does not have a general antibacterial activity, and thereby contradict the existing hypothesis implicating such an effect. The study contributes to the knowledge on the potential impact of breastfeeding on the establishment of a healthy microbiota in infants.

## Findings

### Background and hypothesis

Haptocorrin (previously named transcobalamin I and III, and R-binder) is a glycoprotein, which is present in human milk in relatively large amounts [1,2]. It is characterized by its ability to bind vitamin B12 and other corrinoids [3], and exists in two forms in human milk; apo-haptocorrin, which is unsaturated with vitamin B12, and holo-haptocorrin, which is saturated with vitamin B12 [4]. Human milk contains a much higher concentration of apo-haptocorrin than of holo-haptocorrin [1,2], but the role for the high amount of apo-haptocorrin in human milk is unknown. Furthermore, the function of haptocorrin in different body fluids, i.e. secreted from exocrine glands or released by erythrocytes and leucocytes, remains unknown. However, the current concept is that haptocorrin in adults is responsible for transporting dietary vitamin B12 through the stomach to the intestine. Haptocorrin is believed to be degraded by the intestinal enzymes and the vitamin B12 released from the protein is subsequently bound to intrinsic factor and absorbed in the distal part of ileum [5]. It has been proposed that haptocorrin contributes to the transport and absorption of vitamin B12 in infants while the gastric secretion of IF is not sufficiently developed [6]. On the other hand, the high amount of apo-haptocorrin in human milk has lead to suggestions that haptocorrin, by withholding vitamin B12, exhibits antimicrobial effects in the intestine and thereby potentially protects the infant against pathogenic bacteria [1,7]. Additionally, we speculate that an antibacterial (protective) function of haptocorrin could explain its presence in various body fluids.

The possible effect of haptocorrin on bacteria has been discussed since the late 1970's [1,7-9]. However, only a very limited number of studies addressing such an effect have been performed, and these studies have focused on the effect of haptocorrin on *E. coli *[8-10]. Samson *et al *demonstrated that apo-haptocorrin, derived from human milk, inhibits the growth of the vitamin B12-dependent *E. coli *N.C.I.B. 8134 [8]. This effect was shown to be diminished by pretreatment with trypsin [9]. The gastrointestinal tract of infants differs from the adult gut in terms of higher pH and lower trypsin activity [11-14], thus haptocorrin may still be functional in the upper part of the intestine during infancy. Indeed, it has been demonstrated that porcine haptocorrin is not degraded *in vitro *when subjected to an environment simulating the gastrointestinal tract of infants [10].

Previous studies have shown that both apo-and holo-haptocorrin inhibits the growth of a single enteropathogenic *E. coli O127 *strain (EPEC) [10], thereby suggesting that the haptocorrin moity rather than its ability to bind cobalamin inhibits the growth of pathogens [15]. However, to date no study has systematically investigated the effect of haptocorrin (saturated or unsaturated) on a panel of bacteria.

Recently, the importance of the first colonizing bacteria in infants for the development of a healthy immune system has been highlighted [16,17], and it is known that the microbiota of breastfed infants differ from that of formula fed infants [18]. We hypothesized that haptocorrin from human milk, by inhibiting the growth of certain bacteria and thereby favoring the growth of other bacteria, may influence the initial colonization in breastfed infants. We therefore performed the first systematical study of the effect of haptocorrin on a panel of 34 commensal and pathogenic bacteria to which infants may be exposed, and conclude that human unsaturated haptocorrin had no general antibacterial effect, but was seen to inhibit the growth of *Bifidobacterium breve*, which is commonly found in the intestinal tract of neonates.

## Materials and methods

### Biochemical methods

The concentration of vitamin B12, in BHI and MRS media, was analyzed employing the *Cobas 6000 E *immunoassay system and the analytical kit supplied by the manufacturer (Roche). Unsaturated vitamin B12 binding capacity (UB12BC) in BHI and MRS was measured as described previously [19]. The vitamin B12 content and the UB12BC of *Clostridium difficile *media were not measured due to lack of the liquid form of the specific agar media used in the well-diffusion assay.

### Human milk

Human donor milk, collected at Randers Hospital, Denmark was centrifuged at 10,000 g, 4°C, 15 min. It contained 70 nM haptocorrin and 0.85 nM cobalamin (holo-haptocorrin).

Haptocorrin-free human milk was prepared by affinity purification using polyclonal antibodies against human haptocorrin [3] covalently linked to protein A/G agarose according to instructions of the manufacturer (Santa Cruz). > 99.9% of the haptocorrin was removed, as controlled by ELISA and by measurement of UB12BC. Milk with and without haptocorrin was used without further dilution. The samples were stored at -20°C until use.

### Haptocorrin

Porcine holo-haptocorrin (saturated with vitamin B12) purified from gastric extracts [20] was employed. The protein was diluted in 5 mM phosphate buffer pH 7 to a final concentration of 250 nM. Human haptocorrin unsaturated with vitamin B12 was purified from human donor milk essentially as described previously [21]. The milk originated from a complete lactation from one donor. In brief, 100 mL milk was absorbed to 12 mL vitamin B12-sepharose at room temperature with gentle agitation over night. Subsequently, the B12-sepharose was applied to a glass column. After washing with 500 mL 50 mM Tris-HCl pH 7, 150 mL 50 mM TrisHCl with 0.5 M NaCl pH 7, and 100 mL H_2_O, the absorbed haptocorrin was eluted with 3 × 20 mL 5 M guanidine-HCl each eluted during 20-30 min. The eluates were pooled and dialysed against excess demineralised H_2_O for > 48 h at 4°C. After lyophilization, the protein was redissolved in 5 mM phosphate buffer pH 7 to a final haptocorrin concentration of 250 nM as determined by ELISA, and as judged by UB12BC analysis, > 97% occurred as unsaturated haptocorrin. All haptocorrin samples were filter sterilized (0.22 μm) and stored at -20°C prior to use.

### Bacterial strains and growth media

The 34 selected bacterial strains (Table 1) are either commensal in the stomach or intestine and among the first colonizing bacteria in infants, or are probiotic or pathogenic strains that infants are commonly exposed to [22-26]. The selected strains include 3 listeria, 12 escherichia (11 enteropathogenic species), 7 *Salmonella *strains, 3 bifidobacteria, 2 lactobacilli, 1 *Clostridium*, 2 staphylococci, 2 enterococci, and 2 streptococci. Lactobacilli were cultivated in de Man, Rogosa, Sharpe (MRS) bouillon media, and for well-diffusion assay grown on MRS agar media. *Clostridium difficile *were cultivated in a specific liquid medium (CM81, supplied by Oxoid) and for well-diffussion assay grown on a specific agar medium (Agar Base CM601 with agar supplement SR96, supplied by Oxoid). All other bacteria were cultivated in Brain Heart Infusion (BHI) bouillon media and for well-diffusion assay grown on BHI agar medium. Bifidobacteria, lactobacilli and *Clostridium difficile *were anaerobically incubated for 48 hours, while the rest of the bacteria were aerobically incubated at 24 hours. All strains were incubated at 37°C.

**Table 1:** Effect of human milk, haptocorrin free human milk, apo-haptocorrin and holo-haptocorrin on the growth of selected bacterial strains

Species	Strain and source [reference] ^a, b, c, d^	Human milk	Haptocorrin free human milk^g^	Apo-haptocorrin^h^	Holo-haptocorrin^i^
*Bifidobacterium breve*	DSM no 20091	**+**		**-**	NE
*Bifidobacterium infantis*	DSM no 20088	NE		NE	NE
*Bifidobacterium bifidum*	DSM no 20215	**+**		NE	NE
*Lactobacillus acidophilus*	DSM no 20079	**+**		NE	NE
*Lactobacillus johnsonii*	DSM no 10533	**+**		NE	NE
*Clostridium difficile*	DSM no 1296	NE		NE	NE
*Staphylococcus epidermidis*	DSM no 20044	**+**		NE	NE
*Staphylococcus aureus*	ATCC 29213	NE		NE	NE
*Enterococcus faecalis*	ATCC 29212	NE		NE	NE
*Enterococcus faecium*	CCUG 542 T	NE		NE	NE
*Streptococcus thermophilus*	E2; Tosi *et al*^f^	**-**	**-**	NE	NE
*Streptococcus agalactiae*	CCUG 4208 T	NE		NE	NE
*Escherichia coli*	ATCC 35218	NE		NE	NE
*Escherichia coli *	O157, wild type^e^	NE		NE	NE
*Escherichia coli *	O103:H2, wild type^e^	*+*		NE	NE
*Escherichia coli *	O26:H11, wild type^e^	NE		NE	NE
*Escherichia coli *	O145:H^-^, wild type^e^	**+**		NE	NE
*Escherichia coli *	O26:H^-^, wild type^e^	NE		NE	NE
*Escherichia coli *	O126:H20, wild type^e^	NE		NE	NE
*Escherichia coli *	O128ab:H^-^, wild type^e^	**+**		NE	NE
*Escherichia coli *	O128ab:H2 (1), wild type^e^	**+**		NE	NE
*Escherichia coli *	O128ab:H2 (2), wild type^e^	**+**		NE	NE
*Escherichia coli *	O111:H^-^, wild type^e^	**+**		NE	NE
*Escherichia coli *	O127a:H^-^; DSM no 8702	**+**		NE	NE
*Listeria monocytogenes *	LO28;	**-**	**-**	NE	NE
*Listeria monocytogenes *	4446; Larsen *et al*, 2002^f^	**-**	**-**	NE	NE
*Listeria monocytogenes*	7291; Larsen *et al*, 2002^f ^	**-**	**-**	NE	NE
*Salmonella *Infantis	(1), wild type^e^	NE		NE	NE
*Salmonella *Infantis	(2), wild type^e^	NE		NE	NE
*Salmonella* Enteritidis	(1), wild type^e^	**+**		NE	NE
*Salmonella* Enteritidis	(2), wild type^e^	**+**		NE	NE
*Salmonella *Typhimurium	DT104	NE		NE	NE
*Salmonella *Typhimurium	DT120	NE		NE	NE
*Salmonella *Typhimurium	SL1344	NE		NE	NE

Effect of human milk, haptocorrin free human milk, unsaturated human haptocorrin (250 nM), and saturated porcine haptocorrin (250 nM) respectively, assessed by comparing the growth in corresponding well-diffusion assays with growth in well-diffusion assays with added phosphate buffer (negative control) and added erythromycin (positive control). The results represent three independent experiments giving the same result.

+ indicates increased growth, e.g. increased density of bacteria around the wells (as compared to buffer control)

-indicates visible inhibition (minimum 5 mm clearance zone)

NE: No effect

^a ^Number in parentheses refers to different isolates.

^b ^All DSM strains were obtained from the German Collection of Microorganisms and Cell Cultures.

^c ^All ATCC strains were obtained from the American Type Culture Collection.

^d ^All CCUG strains were obtained from the Culture Collection, University of Göteborg.

^e ^Isolated by the National Food Institute, Denmark.

^f ^Source of strain.

^g ^Assays with addition of haptocorrin free human milk were only conducted for strains inhibited by human milk.

^h ^Apo-haptocorrin was derived from human milk.

^i ^Holo-haptocorrin was derived from porcine gastric juice.

### Well-diffusion assays

The applied well-diffusion assay, which previously has proven useful to screen for bacteriocin activity [27], is suitable when only small amounts of samples are available, and sufficiently sensitive to reveal effects of biological relevance. The assay was a modification of the procedure described by Fuente-Salcido et al.

BHI, MRS or specific *Clostridium difficile *media with agar (2%) was melted and tempered to 50°C. One single cell colony was suspended in 1 mL of the corresponding bouillon (MRS, BHI or a specific *Clostridium difficile *liquid media (CM81)) and mixed with 400 mL agar media, which was plated in 20 agar plates and allowed to gelatinize. A well, 7 mm in diameter, was made in each agar plate and 20 μL of test sample (human milk, haptocorrin-free human milk, porcine holo-haptocorrin and human apo-haptocorrin), 20 μg mL^-1 ^erythromycin (positive control for inhibition) or 5 mM phosphate buffer pH 7 (negative control for inhibition) was added into the well. Only strains inhibited by human milk were subsequently tested with haptocorrin-free human milk. Plates were incubated as described above observing possible inhibition zones. An inhibition zone of minimally 5 mm was defined as a positive inhibitory effect. Assays were performed in triplicates.

## Results

### Media characteristics

BHI media contained 0.13 nM UB12BC and 0.05 nM vitamin B12, whereas the MRS media contained 0.12 nM UB12BC and 0.44 nM vitamin B12, which is concentrations well below the concentration of added haptocorrin (250 nM). Therefore, it seems highly unlikely that the vitamin B12 content and the UB12BC of the media could influence our results, respectively by saturating apo-haptocorrin or taking over haptocorrin's role in vitamin B12 binding.

### Effect of human milk with and without haptocorrin

The positive control (erythromycin) and the negative control (phosphate buffer) gave the expected results for all 34 strains tested; the positive control caused an inhibition zone around the wells, while the negative control did not.

Human milk inhibited growth of *S. thermophilus *and the pathogenic strains *L. monocytogenes L028, L. monocytogenes 4446 *and *L. monocytogenes 7291 *(Table 1). However, this inhibition was not caused by haptocorrin, since haptocorrin free human milk gave the same result.

In some cases, a visible increased density of the bacteria appeared around the wells, when compared to the growth around the wells only containing buffer. This is indicated as 'increased growth' in Table 1, and was observed around the wells after addition of human milk for *Bifidobacterium breve, B. bifidum, Lactobacillus acidophilus, L. johnsonii, Staphylococcus epidermidis, E. coli O103:H2, E. coli O145:H^-^, E. coli O128ab:H^-^, E. coli 128ab:H2(1), E. coli 128ab:H2(2), E. coli O111:H^-^, E. coli O127a:H^-^, Salmonella *Enteritidis *(1) *and *S*. Enteritidis *(2)*.

### Effect of saturated and unsaturated haptocorrin

Due to limited amounts of human milk derived haptocorrin, available porcine haptocorrin was employed for studies on the effect of the saturated form of the protein. Addition of porcine holo-haptocorrin in the well-diffusion assay showed no inhibition of any of the bacteria. Unsaturated human haptocorrin caused an inhibition for *B. breve*, but not for any of the other bacteria.

## Discussion

In the well-diffusion assay originally developed for assessment of bacteriocin activity, [27], human milk inhibited growth of *S. thermophilus *and the pathogenic strains *L. monocytogenes LO28, L. monocytogenes 4446 *and *L. monocytogenes 7291*. For the latter three, this is consistent with the findings of Lopez-Exposito and coworkers, who ascribed the inhibitory effect to the lysozyme in human milk, possibly in combination with lactoferrin/immunoglobulin A [28]. We were surprised to see an inhibitory effect of human milk on *S. thermophilus*, as this species is widely used in the dairy industry [29], but also this is likely to be attributed to lysozyme/lactoferrin/immunoglobulin A or other antibacterial proteins in human milk [30]. Our experiments with haptocorrin-free human milk revealed that the observed inhibitory effect of human milk on the mentioned bacteria was not caused by haptocorrin, because similar inhibition zones were observed for haptocorrin-free human milk as for human milk with haptocorrin. In addition, no inhibitory effect was observed with either purified porcine holo-haptocorrin or with human apo-haptocorrin on the given strains.

Human milk caused a higher density of certain bifidobacteria, lactobacilli, staphylococci, *E. coli *and *Salmonella *strains around the wells. It is well known that lactic acid bacteria and *E. coli *strains can ferment lactose present in human milk [31]. More surprisingly, increased growth was observed for two (non-lactose fermenting) *Salmonella *Enteritidis strains, which may be ascribed to the oligosaccharide content in human milk.

No observable inhibitory effect of porcine holo-haptocorrin on any of the tested bacterial strains was observed. A study by Adkins & Lönnerdal has shown inhibitory effect of porcine apo-and holo-haptocorrin on the growth of a specific enteropathogenic *E. coli *(EPEC) strain [10]. However, our results suggest that such an inhibitory effect is not general towards all EPEC strains. We tested 11 EPEC strains with porcine holo-haptocorrin and did not observe inhibition of the proliferation of any of these bacteria.

It has previously been suggested that the inhibitory effect of human haptocorrin on bacterial growth depends on saturation with vitamin B12, and that only apo-haptocorrin exhibits inhibitory effects [8]. The inhibitory effect of haptocorrin on bacteria may therefore be a matter of absence of vitamin B12 dependence of the strains.

Unsaturated haptocorrin purified from human milk inhibited the growth of a single strain (*B. breve*) of the 34 included strains. This was highly surprising, since *B. breve *is known to be one of the most common bacteria in the gut of breastfed infants [32]. The inhibitory effect was not observed with saturated porcine haptocorrin. This implies that the degree of saturation affects the inhibitory activity of haptocorrin on bacteria, as suggested by Samson *et al *[8]. Alternatively, the origin of protein isolation (human or porcine) may influence the effect of haptocorrin since the amino acid sequence and the degree of glycosylation varies depending on isolation species [33], but further studies are required to elucidate this.

In spite of the observed effect on *B. breve*, our results suggest that haptocorrin does not have a general antibacterial effect, and thereby contradict the existing hypothesis implicating such an effect [1,7,10,15]. Therefore, we find it unlikely that haptocorrin present in human milk through inhibitory properties alter the composition of the first colonizing bacteria in the intestine of breastfed infants.

## Competing interests

The authors declare that they have no competing interests.

## Authors' contributions

HRJ and MFL carried out the microbiological studies, and drafted the manuscript. DLL carried out the analysis related to haptocorin, and participated in the study design and the drafting of the manuscript. JBA contributed to and supervised the microbiological study design. EN and TRL conceived of and initiated the study and participated in its design and interpretation as well as in the drafting of the manuscript. All authors read and approved the final manuscript.
